# Pyrvinium Pamoate Alone and With Gemcitabine Exhibits Anti‐Pancreatic Cancer Activity in 2D and 3D Cell Culture Models

**DOI:** 10.1111/jcmm.70222

**Published:** 2024-12-04

**Authors:** Karabo Serala, Jinming Bai, Sharon Prince

**Affiliations:** ^1^ Department of Human Biology University of Cape Town, Observatory Cape Town South Africa

**Keywords:** drug combinations, drug repurposing, gemcitabine, pancreatic cancer, pyrvinium pamoate

## Abstract

Pancreatic cancer is an intractable disease with the worst prognosis of all common cancers. The treatment regimens currently used for pancreatic cancer do not significantly impact patient survival, and therefore, effective treatment strategies are urgently needed. Drug repurposing, which identifies new indications for existing and approved drugs, has proven to be a desirable approach to anti‐cancer drug discovery. Indeed, the antihelminthic drug, pyrvinium pamoate, has shown promise as an anti‐pancreatic cancer drug. However, the only mechanism of action ascribed to this has been its ability to inhibit mitochondrial function. This study showed, using pancreatic cancer 2D cell cultures and 3D spheroids, that pyrvinium pamoate exhibited short‐ and long‐term cytotoxicity, inhibited epithelial‐to‐mesenchymal transition and cell invasion and migration. Mechanistically, pyrvinium pamoate induced DNA damage, inhibited stemness markers and the PI3K/AKT cell survival pathway, triggered an S‐phase cell cycle arrest and induced apoptotic and autophagic cell death. Importantly, pyrvinium pamoate acted synergistically with the first‐line drug, gemcitabine, in 2D and 3D pancreatic cancer cell culture models. This study provides evidence that pyrvinium pamoate is effective as a single agent and in combination with gemcitabine for the treatment of pancreatic cancer.

## 1 Introduction

Pancreatic cancer (PC) has the worst prognosis of all common cancers, and its worldwide incidence is continuously increasing [1]. The two major PC subtypes are pancreatic ductal adenocarcinoma (PDAC) and pancreatic neuroendocrine tumours (PNETs), with PDAC being the more aggressive subtype and accounting for 90% of all PC cases [2, 3]. At present, surgery is the only curative option for PDAC; however, only 15%–20% of patients present with resectable disease, and of these, a large proportion are not eligible for surgical resection due to immense vascular involvement [4]. The remaining 80% of patients present with either locally advanced or metastatic disease [1]. Clinical care for these patients is through systemic chemotherapy, with the first lines of therapy being gemcitabine (GEM), GEM plus nab‐paclitaxel or FOLFIRINOX [5]. However, these treatment modalities do not significantly impact patient survival due to tumour drug resistance and recurrence, as well as their high toxicity profiles. Developing new drugs to overcome these limitations is challenging, as the drug development pipeline is time‐consuming, risky and expensive [6]. Drug repurposing has therefore gained traction as an alternative approach to anti‐cancer drug discovery [7]. The rationale for this is that the candidate drugs have well‐documented and established pharmacokinetic, pharmacodynamic, dosing and toxicity profiles that accelerate their clinical development [6].

Pyrvinium pamoate (PP) is a small, fluorescent and lipophilic compound that was first described in the 1940s for use as a dye, fluorescent probe and antihelminthic drug [8]. Although the development of more effective anti‐parasitic agents halted its use as an antihelminthic, PP remained clinically relevant. This was due to its potential to target other disease‐causing organisms as well as its ability to reduce toxicity, promote wound repair and inhibit fibrotic tissue development [9]. Of late, PP has gained significant attention in cancer research. Indeed, several studies have reported on its anti‐cancer activities, which involve its ability to inhibit the Wnt signalling pathway and mitochondrial function [8]. In the context of PC, Esumi et al. [10] showed that PP is highly toxic to serum‐starved PANC‐1 cells and can clear PANC‐1 xenografts in vivo. Tomitsuka, Kita, and Esumi [11] compared its effects under hypoxic‐hypoglycaemic conditions, which mimics the tumour microenvironment (TME), and normoxia–normoglycaemic conditions and found that in PDAC, PP inhibits mitochondrial energy metabolism through the inhibition of the NADH‐fumarate reductase (NADH‐FR) system. Schultz et al. [12] recently demonstrated that PP exerts its effects in PDAC not only through specific inhibition of NADH‐FR but also by inducing a more widespread inhibition of mitochondrial function. Collectively, these studies revealed the potential of PP as a TME‐specific anti‐cancer drug. The efficacy of drugs currently used to treat PDAC, including GEM, is negatively affected by the dense and desmoplastic PDAC TME [13]. Consequently, combining PP and GEM may provide an effective treatment option for PDAC.

This study showed that PP inhibited the viability and survival of PDAC 2D cell cultures and 3D spheroids. These effects were mediated through the inhibition of the PI3K/AKT pathway and induction of apoptotic and autophagic cell death. Furthermore, PP inhibited stemness markers, EMT, cell migration and invasion of these cell culture models. Importantly, low concentrations of PP and GEM exhibited synergistic effects and their combination was more effective at exerting anti‐PC activities compared to their use as single agents. Overall, this study extends our understanding of PP's anti‐cancer activities and provides further evidence supporting its use as a single agent or in combination with GEM for the treatment of PC.

## 2 Materials and Methods

### 2.1 Cell Culture

PANC‐1 and CFPAC‐1 PDAC cell lines were purchased from the American Type Culture Collections and maintained in Dulbecco's modified Eagle's medium (DMEM) and Iscove's modified Dulbecco's medium (IMDM) (Gibco, Life Technologies/Thermo Fisher Scientific, USA), respectively. The media were supplemented with 10% heat‐inactivated foetal bovine serum (FBS) and 1% penicillin and streptomycin. Cells were maintained at 37°C in a 95% air and 5% CO_2_‐humidified incubator.

### 2.2 Treatments

PP and GEM were purchased from Sigma Aldrich (Missouri, USA), dissolved in dimethyl sulfoxide (DMSO) to a final concentration of 5 mM and stored at −20°C with limited freeze/thaw cycles. The drugs were diluted in supplemented media to achieve the desired final concentration(s), and the percentage of DMSO in the highest drug concentration was used as a vehicle control.

### 2.3 Cell Viability Assay

Cells were seeded in 96‐well plates and treated for 72 h with increasing concentrations of PP or GEM followed by the MTT assay as previously described [14].

### 2.4 Clonogenic Assays

Cells were treated for 48 h with PP or GEM, and clonogenic assays were performed as previously described [14].

### 2.5 Cell Cycle Analyses

Cells were treated for 72 h with PP or GEM, and cell cycle analysis was performed as previously described [14].

### 2.6 Immunofluorescence

Immunofluorescence was performed as previously described [15]. Briefly, cells were treated with PP or GEM for 72 h and incubated with primary antibodies against γH2AX and LC3 (Table S1). Secondary antibody‐only controls were included in the experiments to remove the background signal. Images were acquired using a confocal microscope LSM880, and fluorescence quantification was performed.

### 2.7 Western Blotting

Total proteins were harvested from PP‐ or GEM‐treated cells, and Western blotting was performed as previously described [16]. The antibodies and concentrations used are shown in Table S2. Densitometric readings were calculated as a ratio of the protein of interest/β‐actin and normalised to either the vehicle control or first lane with a band.

### 2.8 Apoptosis Assay

PP‐ or GEM‐treated cells were fixed with 3:1 methanol: acetic acid and then stained with 1 μg/mL of ethidium bromide and 1 μg/mL of acridine orange for 10 mins in the dark. Cells were imaged under a fluorescence microscope (EVOS M5000 Imaging System, Thermo Fisher Scientific, USA).

### 2.9 Transwell Invasion Assay

PP‐ or GEM‐treated cells were harvested, and 1 × 10^5^ cells were resuspended in media with 1% FBS and replated in transwell inserts coated with Matrigel (CLS354234; Merk, USA). The inserts were placed in 12‐well plates with media with 10% FBS and incubated for 20 h for cell invasion to occur. The non‐invaded cells were removed using cotton swabs, and the invaded cells were fixed with 3.7% paraformaldehyde, stained with 0.5% crystal violet, and imaged under an inverted light microscope.

### 2.10 In Vitro 2D Scratch Motility Assays

The anti‐migratory effects of PP or GEM were investigated as previously described [14].

### 2.11 Spheroid Growth Assays

Cells were plated at 5000 cells/well in 96‐well plates coated with 1.2% agarose to prevent cell adhesion and incubated for 6 days for compact spheroid formation. Once formed, the spheroids were treated with PP or GEM for 6 days, imaged, and their growth rates were calculated by dividing the areas of the spheroids on day 6 by their respective areas on day 0.

### 2.12 Spheroid Viability Assay

The effects of PP and GEM on spheroid viability were assessed by staining with 2 μM calcein‐AM (C1430; Invitrogen, USA). The stained spheroids were imaged under a fluorescence microscope. The calcein‐AM fluorescence intensity was measured using ImageJ, normalised to the spheroid areas, and expressed relative to the vehicle‐treated spheroids.

### 2.13 Spheroid Invasion Assay

The spheroids generated as above were transferred to a new 96‐well plate where they were embedded in 1.5 mg/mL of collagen I rat tail matrix (Gibco, A1048301, Thermo Fisher Scientific, USA), treated for 72 h with PP or GEM, and imaged under an inverted light microscope.

### 2.14 Analyses of Drug Combination

Cells were treated for 72 h with ⅛ IC_50_, ¼ IC_50_ or ½ IC_50_ of PP or GEM as single agents and in combination and subjected to MTT assays. The MTT assay data were analysed using the highest single agent (HSA) model synergy and antagonism model by the Combenefit software and the CompuSyn version 1.0 software according to the instructions [17, 18].

### 2.15 Statistical Analysis

Unless stated, data were obtained from at least three independent experimental repeats and analysed by a parametric unpaired *t*‐test using the GraphPad Prism version 8.0 Software. Error bars represent the standard error of the mean (SEM), and significance was accepted at *p < 0.05, **p < 0.01, ***p < 0.001 and ****p < 0.0001.

## 3 Results

### 3.1 PP Exerts Short‐ and Long‐Term Cytotoxicity and Inhibits Stemness Markers in PDAC Cells

**FIGURE 1:**
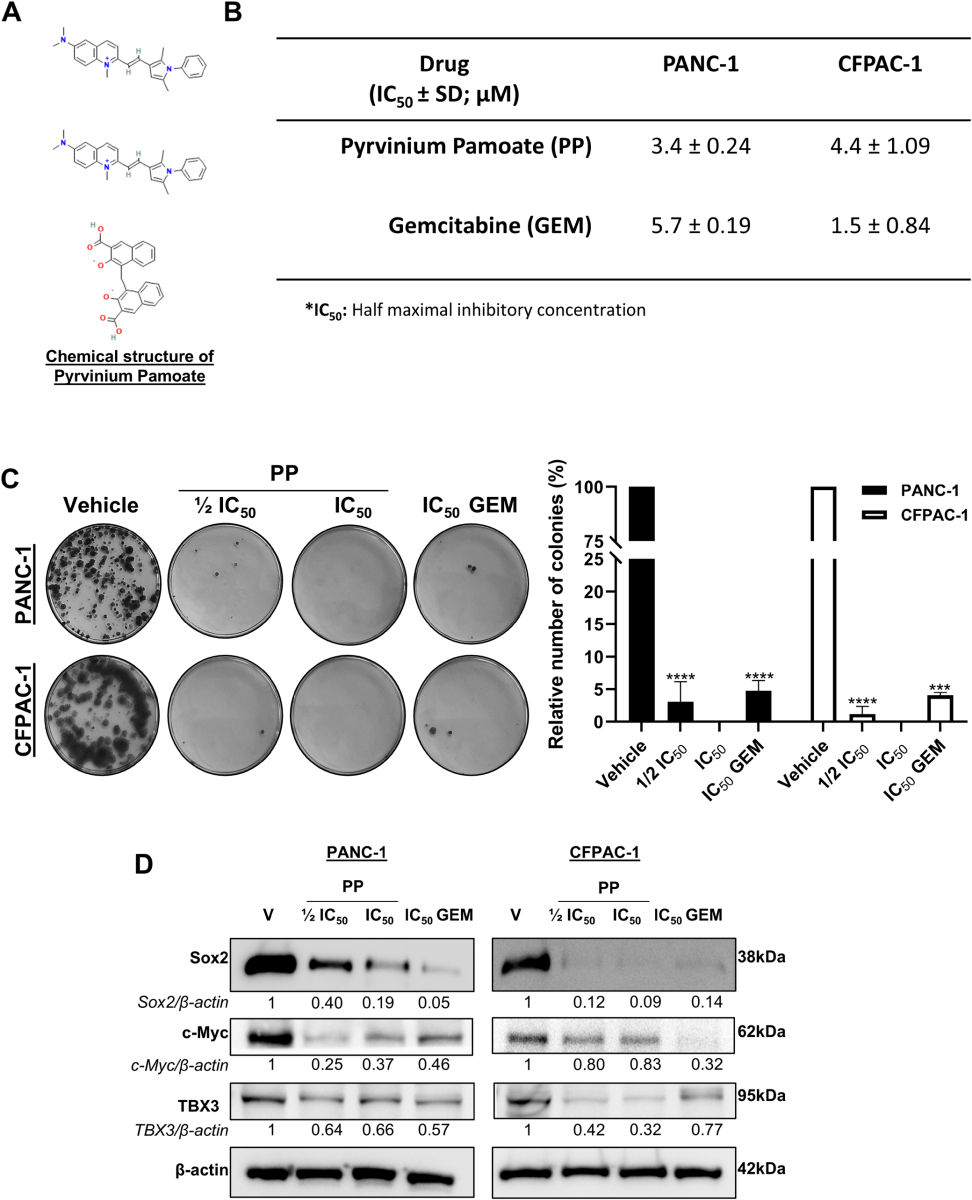
PP exerts short‐ and long‐term cytotoxicity and inhibits PDAC stemness markers in PDAC cells. (A) Chemical structure of PP. (B) MTT cell viability assays of PANC‐1 and CFPAC‐1 cells post 72 h treatment with PP or GEM (*n* = 3). (C) Representative images and quantification of clonogenic assays of PANC‐1 and CFPAC‐1 cells treated as indicated (*n* = 3). (D) Western blot analyses of PDAC stem cell markers sox2, c‐Myc and TBX3 in PDAC cells treated as indicated (*n* = 2). ***p < 0.001 and ****p < 0.0001.

The short‐term cytotoxic effects of PP (Figure 1A) were investigated by treating the PDAC cell lines, PANC‐1 (derived from a primary pancreatic tumour), and CFPAC‐1 (derived from a liver metastasis) for 72 h with the drug, followed by MTT assays. GEM was included as a positive control drug in all experiments. A concentration‐dependent reduction in cell viability was noted for PP, with IC_50_ values of 3.4 ± 0.24 and 4.4 ± 1.09 μM obtained in the PANC‐1 and CFPAC‐1 cell lines, respectively (Figure 1B). It is worth noting that a study by Schultz et al. [12] also determined the IC_50_ values for PP in CFPAC‐1 and PANC‐1 cells and they obtained significantly lower IC_50_ values of 21 and 92 nM, respectively. This may be attributed to their IC_50_ values being obtained after 5 days of treatment and under serum‐free conditions compared to the 3 days of treatment in the supplemented media used in our study. The IC_50_ values obtained for GEM were 5.7 ± 0.19 and 1.5 ± 0.84 μM in the same cell lines, respectively, suggesting that, whereas the PANC‐1 cells were more sensitive to PP, the CFPAC‐1 cells were more sensitive to GEM. Tumour recurrence is common in PDAC, and therefore, the long‐term cytotoxic effects of PP were next investigated using clonogenic assays. The results demonstrated that IC_50_ PP completely abolished colony formation in both PANC‐1 and CFPAC‐1 cells. Moreover, the effects of ½ IC_50_ PP on colony formation were comparable to those observed for IC_50_ GEM, which diminished colony formation by 97.32% and 96.38% in PANC‐1 and CFPAC‐1 cells, respectively (Figure 1C). Importantly, these results correlated with PP and GEM decreasing the levels of the PDAC stemness markers SRY‐Box Transcription Factor 2 (Sox2), cellular myelocytomatosis oncogene (c‐Myc) and T‐BOX transcription factor 3 (TBX3) (Figure 1D) [19, 20, 21].

### 3.2 PP Induces DNA Damage and Triggers Cell Cycle Arrest in PDAC Cells

**FIGURE 2:**
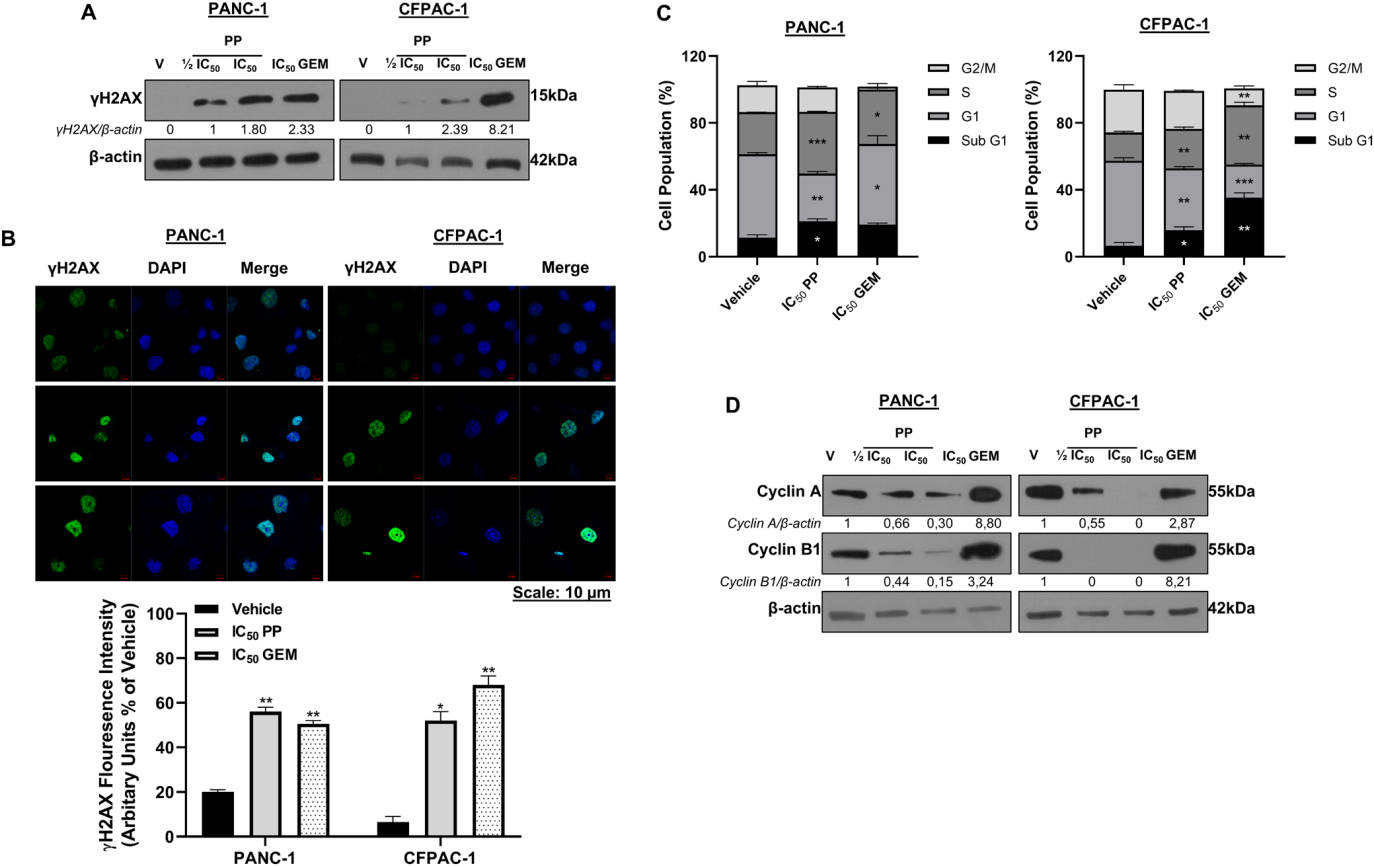
DNA damage induction and cell cycle arrest by PP in PDAC cells. (A) Western blot showing the levels of γH2AX in PDAC cells post 72 h treatment as indicated (*n* = 3). (B) Representative confocal microscope images (×630) and quantitation of γH2AX levels in PDAC cells treated as indicated (*n* = 3). (C) Flow cytometry analysis of cells treated for 72 h with PP or GEM. Graphs represent the mean proportion of cells in each phase of the cell cycle (*n* = 3). (D) Western blot analyses of cycle markers cyclin A and cyclin B1 from cells treated as indicated (*n* = 3). *p < 0.05; **p < 0.01.

To begin to explore the mechanism(s) by which PP exerts its cytotoxicity in PDAC cells, we determined whether it induced DNA damage by measuring the levels of γH2AX by Western blotting and immunocytochemistry. Indeed, the results showed that PP increased the levels of γH2AX in PDAC cells (Figure 2A,B). To determine whether the PP‐induced DNA damage led to cell cycle arrest(s), flow cytometry and Western blotting were performed. The results showed that PP treatment led to an S‐phase cell cycle arrest (Figure 2C), and this correlated with a decrease in levels of cyclin A and cyclin B1 (Figure 2D). Consistent with previous reports, GEM induced an S‐phase arrest in both cell lines as well as a G1 arrest in PANC‐1 cells [22, 23] and this was accompanied by increased cyclin A and cyclin B1 levels. Importantly, there was a significant increase in the sub‐G1 peak of PP‐treated PANC‐1 (112.45%) and CFPAC‐1 (87.46%) cells. Similarly, treatment with IC_50_ GEM led to an increase in PANC‐1 (84.06%) and CFPAC‐1 (322.13%) cells in sub‐G1 (Figure 2C). These findings indicate that PP and GEM induced DNA fragmentation and cell death in PDAC cells [24].

### 3.3 PP Induces Apoptosis in PDAC Cells

**FIGURE 3:**
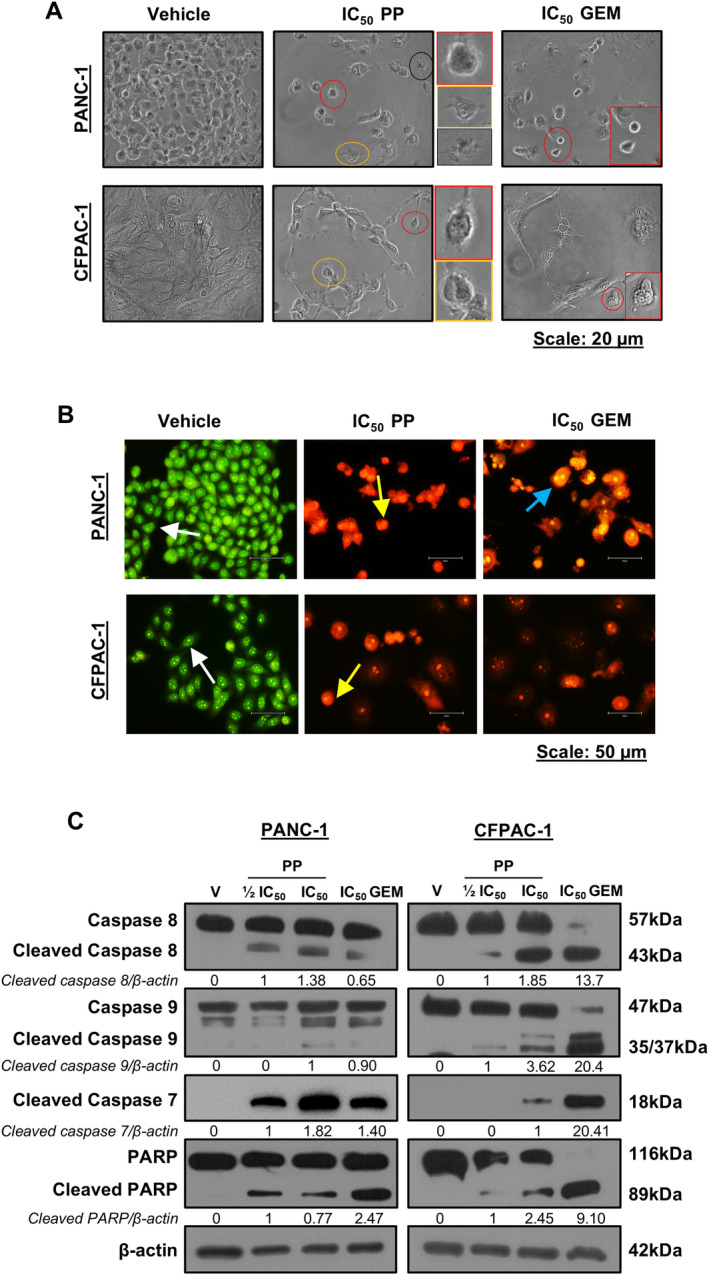
Apoptosis induction by PP in PDAC cells. (A) Representative light microscopy images (×200) showing the impact of PP or GEM on cell morphology (*n* = 3). The coloured circles correspond to the magnified images on the right and indicate morphological features of apoptosis. (B) Representative fluorescence microscope images (40×) showing the effects of PP or GEM on the biochemical features of apoptosis (*n* = 3). The arrows indicate cells in different stages of apoptosis (white arrows: viable cells; yellow arrows: early apoptotic cells, and light blue arrows: late apoptotic cells). (C) Western blot analysis of markers of apoptosis caspase‐8, caspase‐9, cleaved caspase‐7 and PARP in cells treated as indicated (*n* = 3).

To explore if PP induced cell death by apoptosis, we examined the effects of the drug on morphological, biochemical and molecular markers of apoptosis in PDAC cells. Light microscopy images showed that PP‐treated cells exhibited morphological features of apoptosis (Figure 3A). This was confirmed by the acridine orange/ethidium bromide (AO/EB) staining assay, which distinguishes between viable and apoptotic cells based on changes in their membrane permeability. When treated with vehicle, the cells stained green with AO, confirming that they were viable (Figure 3B). In contrast, PP‐treated cells exhibited a combination of yellow (early apoptosis) and reddish/orange (late apoptosis) staining (Figure 3B). Furthermore, treatment with PP led to an increase in cleaved caspase‐8 and cleaved caspase‐9, and this correlated with increased levels of cleaved caspase‐7 and its substrate, PARP (Figure 3C). Similar results were obtained for GEM‐treated cells (Figure 3A–C), which is in line with previous studies that showed that it induces apoptosis in PDAC cells [22, 23, 25].

### 3.4 PP Induces Autophagic Cell Death in PDAC Cells

**FIGURE 4:**
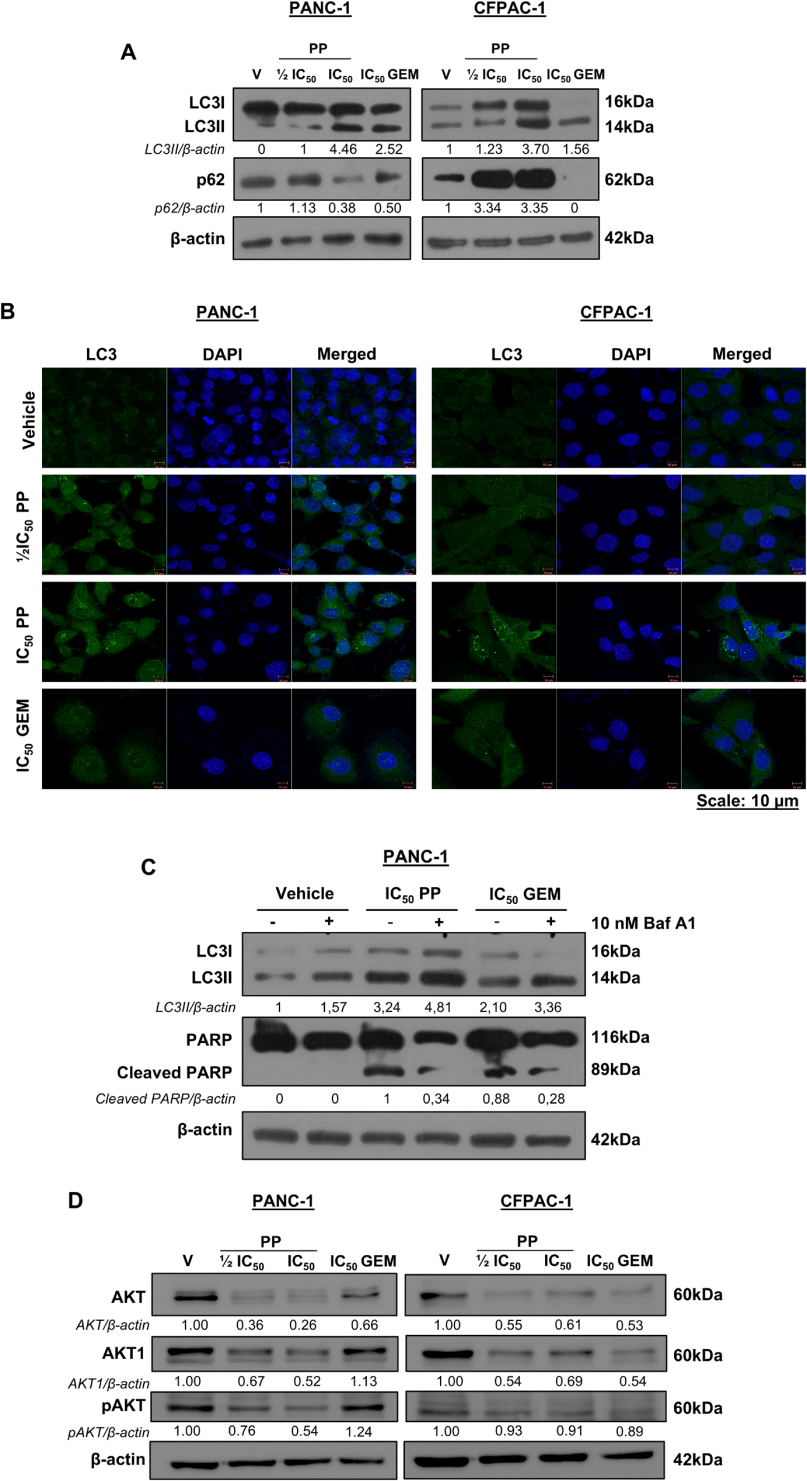
PP induces autophagic cell death and inhibits the PI3K/AKT pathway in PDAC cells. (A) Western blot analysis of molecular markers of autophagy LC3I/II and p62/SQSTM1 in PDAC cells post 72 h treatment as indicated (*n* = 3). (B) Representative confocal microscope images (×630) showing LC3 puncta in cells treated as in (A). (C) Western blot analysis of LC3I/II and PARP in PANC‐1 cells treated for 2 h with 10 nM Bafilomycin A1 followed by 72 h treatment with IC_50_ PP or GEM (*n* = 2). (D) Western blots show AKT, AKT1 and pAKT levels in PDAC cells treated for 72 h as indicated (*n* = 3).

To determine whether PP induced autophagy as an additional mode of cell death, we measured the levels of LC3II in drug‐treated PANC‐1 and CFPAC‐1 cells. Western blotting and immunocytochemistry show that treatment with PP led to a concentration‐dependent increase in LC3II levels (Figure 4A) and LC3 puncta in the cytoplasm of PDAC cells (Figure 4B), respectively. Interestingly, the levels of p62, a cargo protein that gets incorporated and degraded with the autophagosome, decreased in PP‐treated PANC‐1 cells but increased in PP‐treated CFPAC‐1 cells (Figure 4A). These results suggest that PP induced autophagic flux in PANC‐1 cells but not in CFPAC‐1 cells. Treatment with GEM induced LC3II levels and puncta and decreased p62 levels, confirming that it induced autophagic flux in both cell lines (Figure 4A,B). To determine whether the PP‐induced autophagic flux in PANC‐1 cells leads to cell death, the levels of cleaved PARP were measured in the presence and absence of Bafilomycin A1, a chemical inhibitor of autophagy. Figure 4C shows that inhibiting autophagy reduced PARP cleavage in PP‐ and GEM‐treated cells, which confirms that PP and GEM induced autophagic cell death in PANC‐1 cells.

### 3.5 PP Inhibits the AKT Signalling Pathway in PDAC Cells

The AKT signalling pathway is constitutively active in PDAC, and it is well known to inhibit cell death to promote cancer cell survival [26]. Figure 4D confirms that the AKT pathway was constitutively active in the PDAC cells used in this study because vehicle‐treated cells expressed high levels of total AKT, AKT1 (the predominant AKT isoform in PDAC) and pAKT (the activated form of AKT). Importantly, levels of total AKT, AKT1 and pAKT decreased in PP‐treated cells, which indicated that PP inhibited this survival pathway in PDAC cells (Figure 4D). It is worth noting that while GEM also inhibited the levels of these AKT signalling molecules in CFPAC‐1 cells, it did not affect these proteins in PANC‐1 cells. This suggests that PP may be more effective than GEM in preventing the survival of primary PDAC cells.

### 3.6 PP Inhibits PDAC Cell Invasion and Migration

**FIGURE 5:**
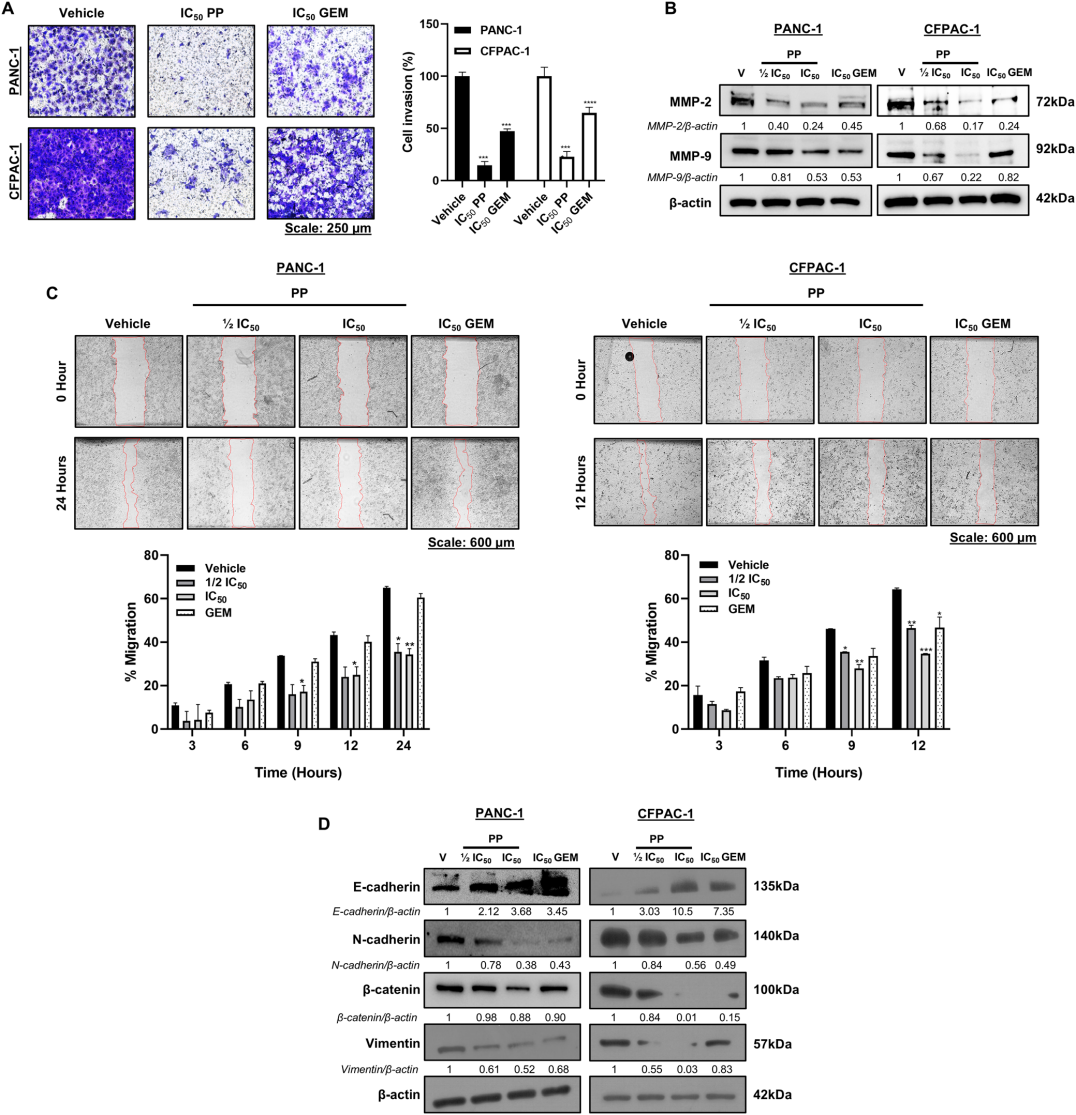
Inhibition of PDAC cell invasion and migration by PP. (A) Representative light microscope images (10×) and quantification of transwell invasion assays of PP‐ or GEM‐treated PDAC cells (*n* = 3). (B) Western blot analyses of cell invasion markers MMP‐2 and MMP‐9 in cells treated as indicated (*n* = 2). (C) Representative light microscope images (4×) of in vitro 2D scratch motility assays post‐treatment with PP or GEM (*n* = 3). (D) Western blot analysis of EMT markers, E‐cadherin, N‐cadherin, β‐catenin and vimentin in cells from (C) (*n* = 2). *p < 0.05, **p < 0.01 ***p < 0.001 and ****p < 0.0001.

Metastasis is the main cause of death in PDAC patients, and therefore, the effects of PP on cell invasion and migration were next assessed. Results from transwell invasion assays showed that PP significantly inhibited the invasiveness of PANC‐1 and CFPAC‐1 cells by 85.15% and 76.98%, respectively (Figure 5A). Notably, PP was more effective than GEM, which inhibited the invasion of the same cell lines by 52.65% and 35.00%, respectively. Consistent with these findings, treatment with PP decreased the levels of the cell invasion markers MMP‐2 and MMP‐9 more than GEM in both PDAC cell lines (Figure 5B). In vitro 2D scratch motility assay results revealed that PP also significantly decreased the migratory abilities of PANC‐1 and CFPAC‐1 cells (Figure 5C). In these assays, GEM was found to not affect PANC‐1 migration and was less effective than PP at inhibiting CFPAC‐1 migration. Cell invasiveness and migration are preceded by EMT and consistent with the results shown in Figure 5A–C; treatment with PP led to an increase in the epithelial marker, E‐cadherin, and a decrease in the mesenchymal markers, N‐cadherin, β‐catenin and vimentin (Figure 5D). Taken together, the data showed that PP inhibited PDAC cell invasiveness and migration probably through the inhibition of EMT, and that it was more effective in doing so than GEM.

### 3.7 PP Inhibits PDAC Spheroid Growth, Viability and Invasiveness

**FIGURE 6:**
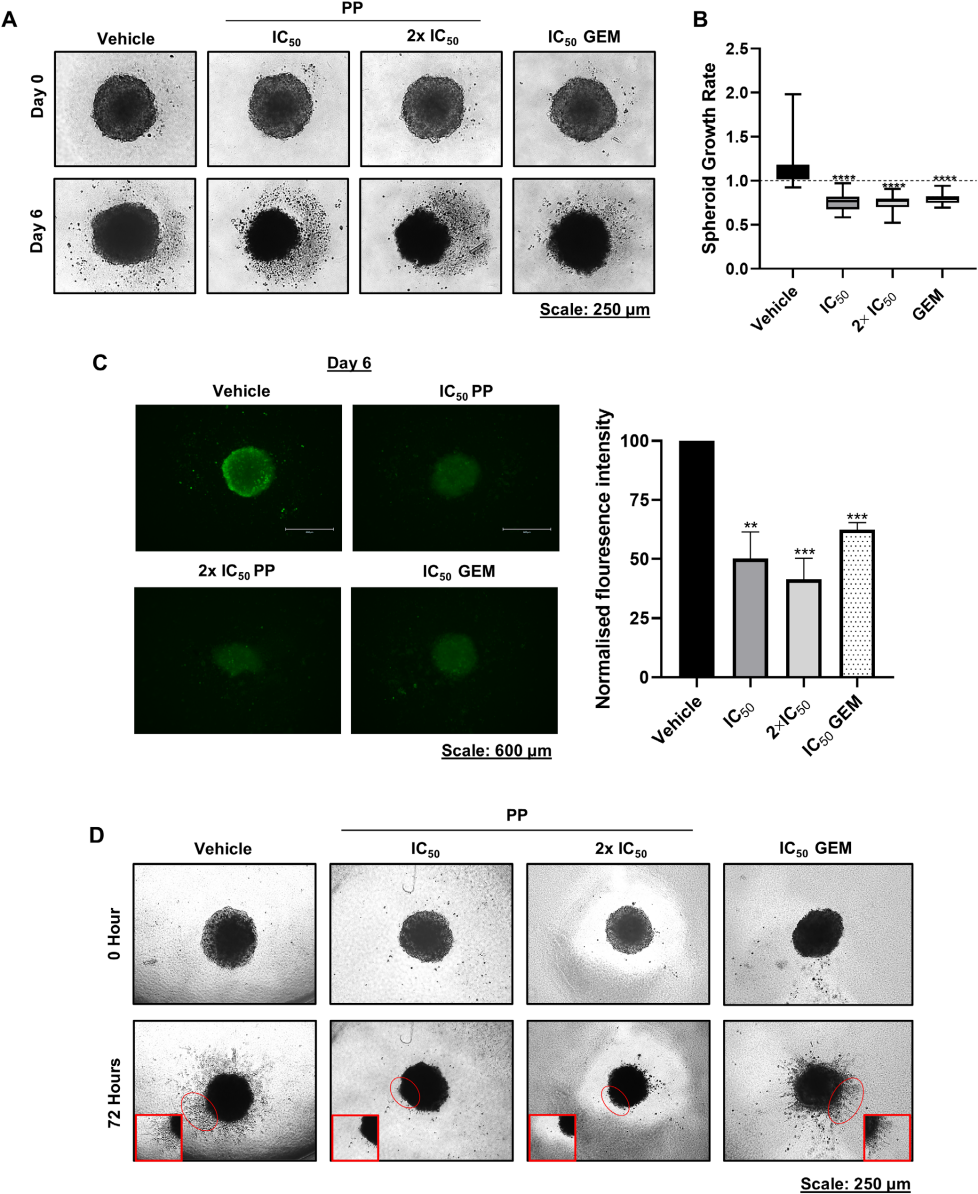
PP inhibits PDAC spheroid growth, viability and invasiveness. (A) Representative light microscope images (10×) of PP‐ or GEM‐treated PDAC spheroids. (B) Quantitation of the growth rate of the spheroid in (A) (*n* = 4). (C) Representative fluorescence microscope images (10×) of the spheroids in (A) stained with calcein‐AM. The accompanying graph shows the mean calcein‐AM fluorescence intensity. (D) Representative light microscope images (4×) of the invasion of PP‐ or GEM‐treated PDAC spheroids (*n* = 3). The red circles correspond to the magnified views on the bottom corners. **p < 0.01 ***p < 0.001 and ****p < 0.0001.

Compared to 2D cell culture models, 3D spheroids more accurately recapitulate the architecture of tumours in vivo [27], and therefore, the cytotoxic and anti‐invasive effects of PP were next validated and compared to GEM in PDAC spheroids. The results showed that compared to vehicle‐treated spheroids, treatment with PP or GEM disintegrated and reduced the size of PDAC spheroids and there were no significant differences in their effects (Figure 6A,B). The effect of PP and GEM on the viability of PDAC spheroids was further assessed using the calcein‐AM fluorescent dye, which is retained and cleaved into a fluorescent product by live cells. Analysis of the calcein‐AM fluorescence intensity revealed that IC_50_ PP, 2 × IC_50_ PP and IC_50_ GEM significantly reduced the PDAC spheroid viability by 42.99%, 58.90% and 34.27%, respectively (Figure 6C).

To determine the effect of PP on spheroid invasion, the PDAC spheroids were embedded in collagen I, a component of the extracellular matrix, and treated with the drug. The results showed that while vehicle‐treated spheroids displayed spindles that protruded from their periphery into the collagen I matrix, PP‐treated spheroids showed no spindles (Figure 6D). Notably, GEM‐treated spheroids showed spindles that protruded into the collagen I matrix; however, these spindles were shorter compared to those observed in vehicle‐treated spheroids (Figure 6D). Together, the results showed that PP inhibited PDAC spheroid growth and invasion, and that it was more effective at inhibiting PDAC invasiveness than GEM in our 3D spheroid model.

### 3.8 PP Enhances the Effects of Gemcitabine on DNA Damage and PDAC Spheroid Viability

**FIGURE 7:**
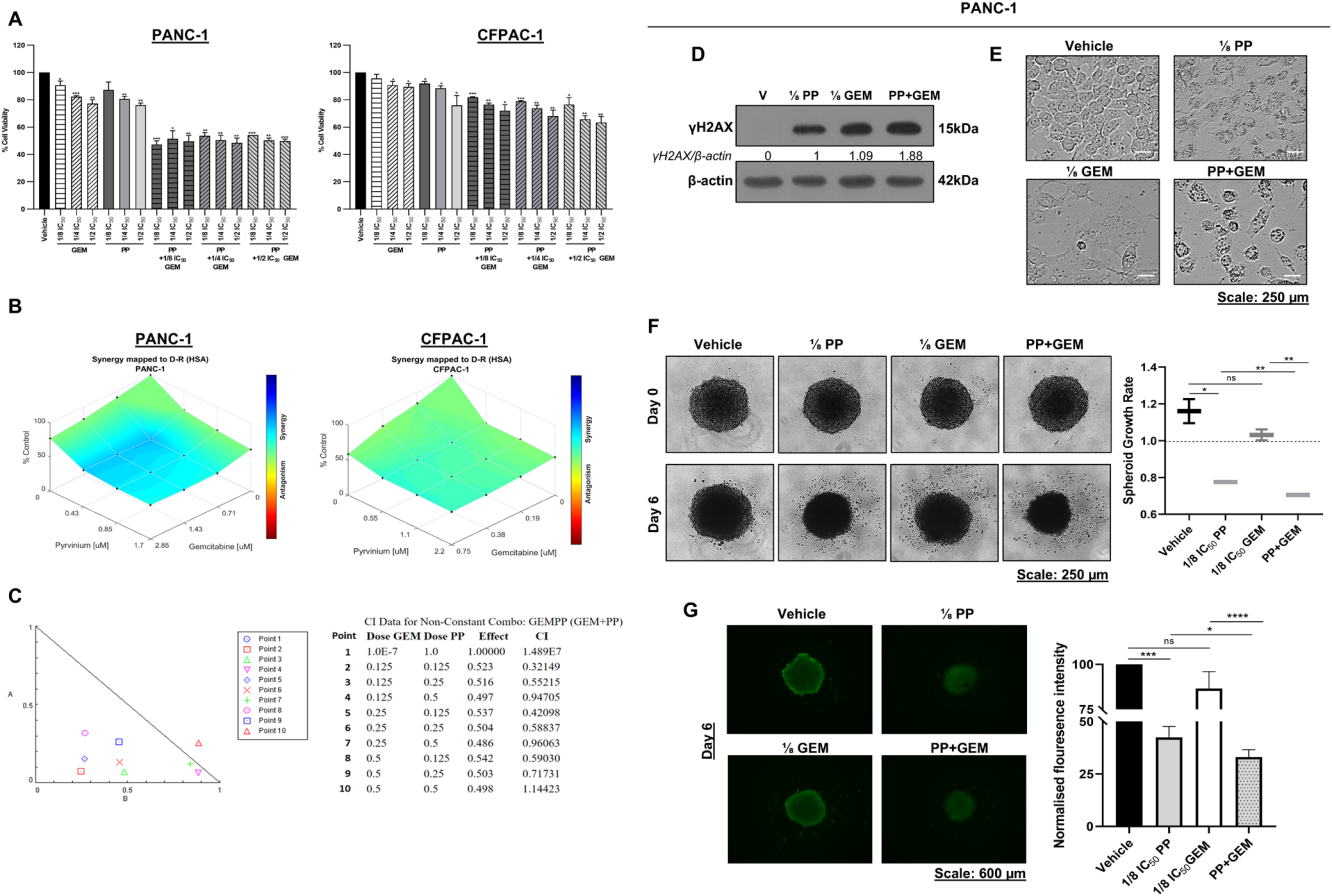
Synergistic effects of PP and GEM on PDAC cell viability, DNA damage and PDAC spheroid viability. (A) MTT assays of PDAC cells post 72 h treatment as indicated (*n* = 3). (B) HSA model surface maps were generated using the Combenefit software. (C) CI plot and values generated using the CompuSyn software. (D) Western blot analysis of γH2AX levels in PANC‐1 treated for 72 h as indicated (*n* = 3). (E) Representative light microscopy images (10×) showing the impact of ⅛ IC_50_ PP or ⅛ IC_50_ GEM or PP + GEM on the morphology of PANC‐1 cells. (F) Representative light microscope images (10×) of PDAC spheroids treated for 6 days as indicated. The accompanying graph indicates spheroid growth rates (*n* = 3). (G) Representative fluorescence microscope images (4×) of the spheroids in (F) stained with calcein‐AM. The accompanying graph shows the mean calcein‐AM fluorescence intensity (*n* = 3). *p < 0.05; **p < 0.01; ***p < 0.001; ****p < 0.0001.

Drug combinations are a cornerstone of cancer therapy [28] because combining two drugs with different mechanisms of action and at lower doses may overcome drug resistance and lead to fewer side effects [29]. Our data suggest that PP and GEM may have different modus operandi and we speculated that PP may improve the anti‐cancer activity of GEM in PDAC patients. To test this, ⅛ IC_50_, ¼ IC_50_ and ½ IC_50_ concentrations of each drug and their combinations were assessed for their effect on PANC‐1 and CFPAC‐1 cell viability using the MTT assay to determine the lowest concentrations at which these drugs acted synergistically. The results showed that all combinations significantly inhibited cell viability more than the drugs on their own in PANC‐1 cells (Figure 7A). To determine the concentrations that result in a synergistic effect, the MTT assay results were analysed using the Combenefit software, and the HSA model synergy and antagonism surface maps were generated. In the surface maps, the blue colour shows synergy, the green colour shows an additive effect and the red colour shows antagonism. As shown in Figure 7B, PP and GEM showed a synergistic effect in PANC‐1 cells but an additive effect in CFPAC‐1 cells. To determine the lowest concentrations of the drugs that gave the highest synergy in PANC‐1 cells, the MTT results were further analysed using publicly available Compusyn software to generate the combination index (CI). In this analysis, CI < 1 shows synergy, CI = 1 shows an additive effect and CI > 1 shows antagonism [30]. The combination of ⅛ IC_50_ PP + ⅛ IC_50_ GEM (hereafter referred to as PP + GEM) showed the strongest synergy with a CI of 0.38 and only the ½ IC_50_ PP + ½ IC_50_ GEM combination showed an antagonistic effect with a CI of 1.14 (Figure 7C). We therefore next compared the ability of PP + GEM to PP (⅛ IC_50_) and GEM (⅛ IC_50_) on their own to induce anti‐cancer activity in PANC‐1 cells. Western blotting showed that all treatments resulted in increased levels of γH2AX but the band for γH2AX was almost twice as intense in the cells treated with PP + GEM (Figure 7D). Light microscopy images show that cells treated with ⅛ IC_50_ PP or ⅛ IC_50_ GEM on their own had a flattened morphology reminiscent of cells undergoing senescence and PP + GEM led to cell detachment and cell shrinkage indicative of cell death (Figure 7E). Furthermore, ⅛ IC_50_ GEM had no significant effect on spheroid growth, reducing it by only 7.69%. In contrast, ⅛ IC_50_ PP on its own significantly inhibited spheroid growth by 36.69%, and this effect was further enhanced to 42.45% when combined with GEM (PP + GEM) (Figure 7F). Analysis of calcein‐AM fluorescence intensity revealed that while ⅛ IC_50_ PP and ⅛ IC_50_ GEM on their own reduced spheroid viability by 13.32% and 58.47%, respectively, combining them (PP + GEM) reduced spheroid viability by 66.86% (Figure 7G). Taken together, the data showed that PP + GEM was more effective than low concentrations (i.e., ⅛ IC_50_) of PP and GEM as single agents in 2D and 3D PDAC culture models.

## 4 Discussion

Drug repurposing has shown great promise in the rapid identification of effective anti‐PDAC drugs, and several FDA‐approved non‐cancer drugs are currently being explored for the treatment of this lethal neoplasm [6]. We were interested in the antihelminthic drug PP because it was shown to inhibit mitochondrial function under conditions that mimic the PDAC TME; however, details of its anti‐PDAC activities and the mechanism(s) involved are not known [10, 11, 12]. In this study, we demonstrated using 2D and 3D PDAC cell culture models that PP induces double‐strand DNA breaks, an S‐phase cell cycle arrest, apoptotic and autophagic cell deaths, and inhibits PDAC stemness markers and the PI3K/AKT survival pathway. Furthermore, we provided important data that show that PP can enhance the anti‐cancer effects of GEM in these cell culture models.

Systemic chemotherapies for PDAC do not significantly improve patient survival due to high toxicity profiles, side effects, tumour drug resistance and the failure to inhibit metastatic spread [5]. Our findings together with those of others who showed that PP induced cytotoxicity in PDAC cells while sparing nonmalignant human pancreatic epithelial cells suggest that it is selective for PDAC cells and may therefore be associated with limited side effects [31, 32, 33]. Moreover, we demonstrate that PP inhibited the colony‐forming ability of PDAC cells, which is significant because post treatment, tumour cells can retain their clonogenicity, ultimately leading to tumour recurrence and relapse [34]. The ability to form clones is a key characteristic of stem cell self‐renewal. Indeed, cancer cells that express high levels of pluripotency markers such as Sox2, Oct4 and Nanog have enhanced clone‐forming abilities [35]. Additionally, c‐Myc has been recognised as a key regulator of stem cell biology, providing a link between malignancy and stemness [36]. In PDAC, modulating the levels of sox2 and c‐Myc pharmacologically disrupts stemness and prevents relapse [21]. Furthermore, TBX3, a c‐Myc downstream target, has been implicated in the expansion of breast cancer stem cells (CSCs) as well as the self‐renewal and stemness of mesenchymal stromal/stem cells, ovarian cancer and PDAC CSCs [19, 27, 37]. Our study showed that PP reduced the levels of Sox2, c‐Myc and TBX3, suggesting that it may inhibit the clonogenic potential of PDAC cells by targeting these cancer stemness markers.

Our findings demonstrate that PP inhibited PDAC spheroid growth, viability and invasion. This is important because compared to 2D cell cultures, 3D spheroids more closely resemble PDAC tumours in terms of their structure and their increased chemoresistance, providing a more accurate drug response [38]. Furthermore, three in four PDAC patients develop recurrence within 2 years after resection, suggesting that even the small proportion of patients who undergo surgical resection harbour micro‐metastasis [5]. Indeed, PDAC cells acquire pro‐metastatic traits that enable them to disseminate and spread before primary tumour formation, resulting in PDAC patients presenting with liver (76%–80%), peritoneum (48%) or lung (45%) metastasis at diagnosis [39, 40]. Among the key regulators of PDAC metastasis is the Wnt/β‐catenin pathway. Upon activation, this pathway leads to the translocation of β‐catenin into the nucleus where it facilitates the transcription of genes that induce EMT, invasion and migration to distant sites [41, 42]. PP is a well‐known inhibitor of the Wnt/β‐catenin pathway [41, 43], suggesting that it may have exerted its anti‐metastatic effects in our PDAC cells, in part, by inhibiting this pathway. Future studies should further explore this potential mechanism.

Understanding the mechanism(s) by which anti‐cancer drugs function may provide insight into the potential mechanisms of tumour drug resistance [44]. This study provided evidence that the anti‐cancer effects of PP occur through mechanisms that may enable it to escape resistance by PDAC cells. PP triggered apoptotic and autophagic cell death, and this is important because anti‐cancer agents that induce more than one form of programmed cell death are more efficacious and less prone to resistance [45]. Furthermore, PP's ability to activate intrinsic and extrinsic apoptotic pathways is significant, as the inactivation of the intrinsic apoptotic pathway is one of the mechanisms by which PDAC cells become resistant to GEM [45, 46]. In addition, our observations that PP induced autophagic cell death are of interest because autophagy is elevated in the late stages of PDAC, where it fuels tumour metabolism to promote tumour growth and drug resistance [47]. Consistent with our data, another antihelminthic drug niclosamide inhibited PARP cleavage in the presence of a late inhibitor of autophagy, Bafilomycin A1, in PDAC cells [48]. Finally, the PI3K/AKT pathway and increased activity of the Wnt/β‐catenin pathway contribute to the mechanisms by which PDAC cells develop drug resistance [49, 50]. PP is well known for its ability to inhibit the Wnt/β‐catenin pathway [41, 43], and our findings that it also inhibits the AKT pathway in PDAC cells are important because the ability of the Wnt/β‐catenin and PI3K/AKT/mTORC1 pathways to compensate for one another is one of the main mechanisms of chemoresistance [51]. Furthermore, inhibiting the PI3K/AKT pathway with wortmannin and LY294002 sensitised PDAC cells to GEM [52] and inhibition of PKB/AKT by wortmannin was shown to promote the anti‐cancer activities of GEM in PDAC mouse models [53]. Taken together, these findings suggest that PP, as an inhibitor of both the Wnt/β‐catenin and AKT pathways, may be effective in overcoming PDAC chemoresistance when combined with GEM.

Combination therapies have proven to be an effective strategy for the management of PDAC. By combining low doses of drugs with distinct mechanisms of action, drug resistance may be overcome, leading to increased efficacy with minimal or no side effects [29]. Most anti‐PDAC combination regimens are centred on GEM because it is the only FDA‐approved monotherapy and several of these including the FDA‐approved GEM plus nab‐paclitaxel have improved patient survival [54]. However, these combinations fail to achieve complete remission and they are associated with high toxicities and a high degree of intrinsic or acquired resistance due to the PDAC TME [55]. Indeed, the PDAC TME limits the delivery of GEM to tumours by downregulating the concentrative and equilibrative nucleoside transporters and enzymes involved in GEM transport and metabolism, respectively [56]. Therefore, less cytotoxic combinations that target both PDAC cells and the TME are urgently needed to combat PDAC. PP was previously shown to be effective under conditions that mimic the PDAC TME [10, 11, 12] and here we showed for the first time that PP can improve the anti‐cancer activities of GEM in PDAC. Combining PP with GEM may therefore lead to significant improvements in PDAC treatment. In this regard, it is worth noting that an ongoing phase I clinical trial (NCT05055323) is investigating the safety and tolerability of PP in early‐stage PDAC patients [57]. Based on our findings, we recommend extending this trial to include the combination of PP with GEM.

## 5 Conclusions

This study provided evidence that PP may be an effective drug that can be repurposed to treat PDAC patients on its own or together with GEM and we revealed novel mechanisms by which PP achieved anti‐PDAC activities. Additional experiments are required to fully characterise and investigate the mechanisms of action of the PP + GEM combination, and their efficacy needs to be validated in vivo, in, for example, PDAC patient–derived xenografts models.

## Author Contributions

**Karabo Serala:** conceptualization (equal), data curation (equal), formal analysis (equal), investigation (equal), methodology (equal), validation (equal), writing – original draft (equal), writing – review and editing (equal). **Jinming Bai:** data curation (equal), formal analysis (equal), investigation (equal), validation (equal), writing – review and editing (equal). **Sharon Prince:** conceptualization (lead), formal analysis (equal), funding acquisition (lead), project administration (lead), resources (lead), supervision (lead), writing – original draft (equal), writing – review and editing (lead).

## Ethics Statement

The authors have nothing to report.

## Conflicts of Interest

The authors declare no conflicts of interest.

## Supporting information


Table S1.
**Table S2**.

## Data Availability

The data presented in this article are available.

## References

[1] J. Chen , H. Zhang , C. Xiu , et al., “METTL3 Promotes Pancreatic Cancer Proliferation and Stemness by Increasing Stability of ID2 mRNA in a m6A‐Dependent Manner,” Cancer Letters 565 (2023): 216222. 37196908 10.1016/j.canlet.2023.216222

[2] P. Rawla , T. Sunkara , and V. Gaduputi , “Epidemiology of Pancreatic Cancer: Global Trends, Aetiology and Risk Factors,” World Journal of Oncology 10 (2019): 10–27. 30834048 10.14740/wjon1166 PMC6396775

[3] L. Kumar , S. Kumar , K. Sandeep , and S. K. S. Patel , “Therapeutic Approaches in Pancreatic Cancer: Recent Updates,” Biomedicine 11 (2023): 1611. 10.3390/biomedicines11061611 PMC10296116 37371705

[4] M. de Scordilli , A. Michelotti , D. Zara , et al., “Preoperative Treatments in Borderline Resectable and Locally Advanced Pancreatic Cancer: Current Evidence and New Perspectives,” Critical Reviews in Oncology/Hematology 186 (2023): 104013. 37116817 10.1016/j.critrevonc.2023.104013

[5] C. J. Halbrook , C. A. Lyssiotis , M. Pasca di Magliano , and A. Maitra , “Pancreatic Cancer: Advances and Challenges,” Cell 186 (2023): 1729–1754. 37059070 10.1016/j.cell.2023.02.014 PMC10182830

[6] L. De Lellis , S. Veschi , N. Tinari , et al., “Drug Repurposing, an Attractive Strategy in Pancreatic Cancer Treatment: Preclinical and Clinical Updates,” Cancers (Basel) 13 (2021): 3946. 34439102 10.3390/cancers13163946 PMC8394389

[7] L. Sleire , H. E. Førde , I. A. Netland , L. Leiss , B. S. Skeie , and P. Ø. Enger , “Drug Repurposing in Cancer,” Pharmacological Research 124 (2017): 74–91. 28712971 10.1016/j.phrs.2017.07.013

[8] A. A. Momtazi‐borojeni , E. Abdollahi , F. Ghasemi , M. Caraglia , and A. Sahebkar , “The Novel Role of Pyrvinium in Cancer Therapy,” Journal of Cellular Physiology 233 (2018): 2871–2881. 28500633 10.1002/jcp.26006

[9] C. W. Schultz and A. Nevler , “Pyrvinium Pamoate: Past, Present, and Future as an Anti‐Cancer Drug,” Biomedicine 10 (2022): 3249. 10.3390/biomedicines10123249 PMC9775650 36552005

[10] H. Esumi , J. Lu , Y. Kurashima , and T. Hanaoka , “Antitumor Activity of Pyrvinium Pamoate, 6‐(Dimethylamino)‐2‐[2‐(2,5‐Dimethyl‐1‐Phenyl‐1H‐Pyrrol‐3‐yl)Ethenyl]‐1‐Methyl‐Quinolinium Pamoate Salt, Showing Preferential Cytotoxicity During Glucose Starvation,” Cancer Science 95 (2004): 685–690. 15298733 10.1111/j.1349-7006.2004.tb03330.x PMC11159109

[11] E. Tomitsuka , K. Kita , and H. Esumi , “An Anticancer Agent, Pyrvinium Pamoate Inhibits the NADH–Fumarate Reductase System—A Unique Mitochondrial Energy Metabolism in Tumour Microenvironments,” Journal of Biochemistry 152 (2012): 171–183. 22528668 10.1093/jb/mvs041

[12] C. W. Schultz , G. A. McCarthy , T. Nerwal , et al., “The FDA‐Approved Anthelmintic Pyrvinium Pamoate Inhibits Pancreatic Cancer Cells in Nutrient‐Depleted Conditions by Targeting the Mitochondria,” Molecular Cancer Therapeutics 20 (2021): 2166–2176. 34413127 10.1158/1535-7163.MCT-20-0652 PMC8859979

[13] Q. Liu , Q. Liao , and Y. Zhao , “Chemotherapy and Tumour Microenvironment of Pancreatic Cancer,” Cancer Cell International 17 (2017): 68. 28694739 10.1186/s12935-017-0437-3 PMC5498917

[14] J. S. Bleloch , A. du Toit , L. Gibhard , et al., “The Palladacycle Complex AJ‐5 Induces Apoptotic Cell Death While Reducing Autophagic Flux in Rhabdomyosarcoma Cells,” Cell Death Discovery 5 (2019): 60. 30701092 10.1038/s41420-019-0139-9 PMC6349869

[15] T. Willmer , J. Peres , S. Mowla , A. Abrahams , and S. Prince , “The T‐Box Factor TBX3 Is Important in S‐Phase and Is Regulated by c‐Myc and Cyclin A‐CDK2,” Cell Cycle 14 (2015): 3173–3183. 26266831 10.1080/15384101.2015.1080398 PMC4825571

[16] S. Prince , T. Wiggins , P. A. Hulley , and S. H. Kidson , “Stimulation of Melanogenesis by Tetradecanoylphorbol 13‐Acetate (TPA) in Mouse Melanocytes and Neural Crest Cells,” Pigment Cell Research 16 (2003): 26–34. 12519122 10.1034/j.1600-0749.2003.00008.x

[17] T.‐C. Chou , “Drug Combination Studies and Their Synergy Quantification Using the Chou‐Talalay Method,” Cancer Research 70 (2010): 440–446. 20068163 10.1158/0008-5472.CAN-09-1947

[18] G. Y. Di Veroli , C. Fornari , D. Wang , et al., “Combenefit: An Interactive Platform for the Analysis and Visualisation of Drug Combinations,” Bioinformatics 32 (2016): 2866–2868. 27153664 10.1093/bioinformatics/btw230 PMC5018366

[19] L. Perkhofer , K. Walter , I. G. Costa , et al., “Tbx3 Fosters Pancreatic Cancer Growth by Increased Angiogenesis and Activin/Nodal‐Dependent Induction of Stemness,” Stem Cell Research 17 (2016): 367–378. 27632063 10.1016/j.scr.2016.08.007

[20] M. Herreros‐Villanueva , J. S. Zhang , A. Koenig , et al., “SOX2 Promotes Dedifferentiation and Imparts Stem Cell‐Like Features to Pancreatic Cancer Cells,” Oncogene 2 (2013): e61. 10.1038/oncsis.2013.23 PMC3759123 23917223

[21] Q. Zheng , J. Tang , A. Aicher , et al., “Inhibiting NR5A2 Targets Stemness in Pancreatic Cancer by Disrupting SOX2/MYC Signalling and Restoring Chemosensitivity,” Journal of Experimental & Clinical Cancer Research 42 (2023): 323. 38012687 10.1186/s13046-023-02883-y PMC10683265

[22] D. Li , Y. M. Jia , P. K. Cao , W. Wang , B. Liu , and Y. L. Li , “Combined Effect of 125 I and Gemcitabine on PANC‐1 Cells: Cellular Apoptosis and Cell Cycle Arrest,” Journal of Cancer Research and Therapeutics 14 (2018): 1476. 30589026 10.4103/jcrt.JCRT_43_18

[23] C. Panebianco , N. Trivieri , A. Villani , et al., “Improving Gemcitabine Sensitivity in Pancreatic Cancer Cells by Restoring miRNA‐217 Levels,” Biomolecules 11 (2021): 639. 33925948 10.3390/biom11050639 PMC8146031

[24] C. Riccardi and I. Nicoletti , “Analysis of Apoptosis by Propidium Iodide Staining and Flow Cytometry,” Nature Protocols 1 (2006): 1458–1461. 17406435 10.1038/nprot.2006.238

[25] S. H. Park , J. H. Sung , E. J. Kim , and N. Chung , “Berberine Induces Apoptosis via ROS Generation in PANC‐1 and MIA‐PaCa2 Pancreatic Cell Lines,” Brazilian Journal of Medical and Biological Research 48 (2015): 111–119. 25517919 10.1590/1414-431X20144293 PMC4321216

[26] Y. Meng , W. Wang , J. Kang , X. Wang , and L. Sun , “Role of the PI3K/AKT Signalling Pathway in Apoptotic Cell Death in the Cerebral Cortex of Streptozotocin‐Induced Diabetic Rats,” Experimental and Therapeutic Medicine 13 (2017): 2417–2422. 28565857 10.3892/etm.2017.4259 PMC5443277

[27] V. Damerell , M. A. Ambele , S. Salisbury , et al., “The c‐Myc/TBX3 Axis Promotes Cellular Transformation of Sarcoma‐Initiating Cells,” Frontiers in Oncology 11 (2022): 801691. 35145908 10.3389/fonc.2021.801691 PMC8821881

[28] R. B. Mokhtari , T. S. Homayouni , N. Baluch , et al., “Combination Therapy in Combating Cancer,” Oncotarget 8 (2017): 38022–38043. 28410237 10.18632/oncotarget.16723 PMC5514969

[29] Y. KalantarMotamedi , R. J. Choi , S. B. Koh , J. L. Bramhall , T. P. Fan , and A. Bender , “Prediction and Identification of Synergistic Compound Combinations Against Pancreatic Cancer Cells,” iScience 24 (2021): 103080. 34585118 10.1016/j.isci.2021.103080 PMC8456050

[30] T.‐C. Chou , “The Combination Index (CI < 1) as the Definition of Synergism and of Synergy Claims,” Synergy 7 (2018): 49–50.

[31] W. Zheng , J. Hu , Y. Lv , et al., “Pyrvinium Pamoate Inhibits Cell Proliferation Through ROS‐Mediated AKT‐Dependent Signalling Pathway in Colorectal Cancer,” Medical Oncology 38 (2021): 21. 33554313 10.1007/s12032-021-01472-3 PMC7868320

[32] A. Wiegering , F. W. Uthe , M. Hüttenrauch , et al., “The Impact of Pyrvinium Pamoate on Colon Cancer Cell Viability,” International Journal of Colorectal Disease 29 (2014): 1189–1198. 25060218 10.1007/s00384-014-1975-y

[33] J. Feng , W. Jiang , Y. Liu , et al., “Blocking STAT3 by Pyrvinium Pamoate Causes Metabolic Lethality in KRAS‐Mutant Lung Cancer,” Biochemical Pharmacology 177 (2020): 113960. 32298693 10.1016/j.bcp.2020.113960

[34] H. Fiebig , A. Maier , and A. Burger , “Clonogenic Assay With Established Human Tumour Xenografts,” European Journal of Cancer 40 (2004): 802–820. 15120036 10.1016/j.ejca.2004.01.009

[35] K. Takahashi and S. Yamanaka , “Induction of Pluripotent Stem Cells From Mouse Embryonic and Adult Fibroblast Cultures by Defined Factors,” Cell 126 (2006): 663–676. 16904174 10.1016/j.cell.2006.07.024

[36] H. Zhang , P. Wang , M. Lu , S. Zhang , and L. Zheng , “c‐Myc Maintains the Self‐Renewal and Chemoresistance Properties of Colon Cancer Stem Cells,” Oncology Letters 17, no. 5 (2019): 4487–4493, 10.3892/ol.2019.10081. 30944638 PMC6444394

[37] Y. Zhang , J. Guo , E. Cai , et al., “HOTAIR Maintains the Stemness of Ovarian Cancer Stem Cells via the miR‐206/TBX3 Axis,” Experimental Cell Research 395 (2020): 112218. 32771526 10.1016/j.yexcr.2020.112218

[38] X. Liu , B. Gündel , X. Li , et al., “3D Heterospecies Spheroids of Pancreatic Stroma and Cancer Cells Demonstrate Key Phenotypes of Pancreatic Ductal Adenocarcinoma,” Translational Oncology 14 (2021): 101107. 33946033 10.1016/j.tranon.2021.101107 PMC8111319

[39] V. B. Joshi , O. L. Gutierrez Ruiz , and G. L. Razidlo , “The Cell Biology of Metastatic Invasion in Pancreatic Cancer: Updates and Mechanistic Insights,” Cancers (Basel) 15 (2023): 2169. 37046830 10.3390/cancers15072169 PMC10093482

[40] M. Miquel , S. Zhang , and C. Pilarsky , “Pre‐Clinical Models of Metastasis in Pancreatic Cancer,” Frontiers in Cell and Developmental Biology 9 (2021): 748631. 34778259 10.3389/fcell.2021.748631 PMC8578999

[41] H. Li , S. Liu , R. Jin , et al., “Pyrvinium Pamoate Regulates MGMT Expression Through Suppressing the Wnt/β‐Catenin Signalling Pathway to Enhance the Glioblastoma Sensitivity to Temozolomide,” Cell Death Discovery 7 (2021): 288. 34642308 10.1038/s41420-021-00654-2 PMC8511032

[42] P. Zhou , Y. Li , B. Li , et al., “NMIIA Promotes Tumour Growth and Metastasis by Activating the Wnt/β‐Catenin Signalling Pathway and EMT in Pancreatic Cancer,” Oncogene 38 (2019): 5500–5515. 30967633 10.1038/s41388-019-0806-6

[43] C. Zhang , Z. Zhang , S. Zhang , W. Wang , and P. Hu , “Targeting of Wnt/β‐Catenin by Anthelmintic Drug Pyrvinium Enhances Sensitivity of Ovarian Cancer Cells to Chemotherapy,” Medical Science Monitor 23 (2017): 266–275. 28090074 10.12659/MSM.901667 PMC5266205

[44] R. C. Cattley and B. R. Radinsky , “Cancer Therapeutics: Understanding the Mechanism of Action,” Toxicologic Pathology 32 (2004): 116–121. 15209411 10.1080/01926230490426507

[45] S. I. Omoruyi , O. E. Ekpo , D. M. Semenya , A. Jardine , and S. Prince , “Exploitation of a Novel Phenothiazine Derivative for Its Anti‐Cancer Activities in Malignant Glioblastoma,” Apoptosis 25 (2020): 261–274. 32036474 10.1007/s10495-020-01594-5

[46] Y. Jia and J. Xie , “Promising Molecular Mechanisms Responsible for Gemcitabine Resistance in Cancer,” Genes & Diseases 2 (2015): 299–306. 30258872 10.1016/j.gendis.2015.07.003 PMC6150077

[47] K. Yamamoto , D. Iwadate , H. Kato , Y. Nakai , K. Tateishi , and M. Fujishiro , “Targeting Autophagy as a Therapeutic Strategy Against Pancreatic Cancer,” Journal of Gastroenterology 57 (2022): 603–618. 35727403 10.1007/s00535-022-01889-1 PMC9392712

[48] J. B. Kaushal , R. Bhatia , R. K. Kanchan , et al., “Repurposing Niclosamide for Targeting Pancreatic Cancer by Inhibiting Hh/Gli Non‐Canonical Axis of Gsk3β,” Cancers (Basel) 13 (2021): 3105. 34206370 10.3390/cancers13133105 PMC8269055

[49] S. Mehra , N. Deshpande , and N. Nagathihalli , “Targeting PI3K Pathway in Pancreatic Ductal Adenocarcinoma: Rationale and Progress,” Cancers (Basel) 13 (2021): 4434. 34503244 10.3390/cancers13174434 PMC8430624

[50] C. Zhou , C. Yi , Y. Yi , et al., “LncRNA PVT1 Promotes Gemcitabine Resistance of Pancreatic Cancer via Activating Wnt/β‐Catenin and Autophagy Pathway Through Modulating the miR‐619‐5p/Pygo2 and miR‐619‐5p/ATG14 Axes,” Molecular Cancer 19 (2020): 118. 32727463 10.1186/s12943-020-01237-y PMC7389684

[51] A. Prossomariti , G. Piazzi , C. Alquati , and L. Ricciardiello , “Are Wnt/β‐Catenin and PI3K/AKT/mTORC1 Distinct Pathways in Colorectal Cancer?,” Cellular and Molecular Gastroenterology and Hepatology 10 (2020): 491–506. 32334125 10.1016/j.jcmgh.2020.04.007 PMC7369353

[52] S. S. W. Ng , M. S. Tsao , S. Chow , and D. W. Hedley , “Inhibition of Phosphatidylinositide 3‐Kinase Enhances Gemcitabine‐Induced Apoptosis in Human Pancreatic Cancer Cells,” Cancer Research 60 (2000): 5451–5455. 11034087

[53] S. S. Ng , M. S. Tsao , T. Nicklee , and D. W. Hedley , “Wortmannin Inhibits pkb/akt Phosphorylation and Promotes Gemcitabine Antitumor Activity in Orthotopic Human Pancreatic Cancer Xenografts in Immunodeficient Mice,” Clinical Cancer Research 7 (2001): 3269–3275. 11595724

[54] F. Lei , X. Xi , S. K. Batra , and T. K. Bronich , “Combination Therapies and Drug Delivery Platforms in Combating Pancreatic Cancer,” Journal of Pharmacology and Experimental Therapeutics 370 (2019): 682–694. 30796131 10.1124/jpet.118.255786 PMC6806650

[55] A. K. Beutel and C. J. Halbrook , “Barriers and Opportunities for Gemcitabine in Pancreatic Cancer Therapy,” American Journal of Physiology‐Cell Physiology 324 (2023): C540–C552. 36571444 10.1152/ajpcell.00331.2022 PMC9925166

[56] J. Natu and G. P. Nagaraju , “Gemcitabine Effects on Tumour Microenvironment of Pancreatic Cancer: Special Focus on Resistance Mechanisms and Metronomic Therapies,” Cancer Letters 216 (2023): 382, 10.1016/j.canlet.2023.216382. 37666293

[57] F. M. Ponzini , C. W. Schultz , B. E. Leiby , et al., “Repurposing the FDA‐Approved Anthelmintic Pyrvinium Pamoate for Pancreatic Cancer Treatment: Study Protocol for a Phase I Clinical Trial in Early‐Stage Pancreatic Ductal Adenocarcinoma,” BMJ Open 13 (2023): e073839. 10.1136/bmjopen-2023-073839 PMC10582846 37848297

