# Supplementary material for: Pyrvinium Pamoate Alone and With Gemcitabine Exhibits Anti‐Pancreatic Cancer Activity in 2D and 3D Cell Culture Models
Source: J Cell Mol Med. 2024 Dec 4;28(23):e70222. doi: 10.1111/jcmm.70222 (PMC11617115; doi:10.1111/jcmm.70222)
Supplement: Supplementary file 1 — Table S1. Table S2. [file JCMM-28-e70222-s001.docx]

**Table S1: Immunofluorescence antibody list**

| **Antibody** | **Supplier** | **Dilution** |
| --- | --- | --- |
| Phospho-Histone H2A.X (Ser139) | Cell Signaling Technology | 1:100 |
| Microtubule-associated protein light chain 3 (LC3) | Cell Signaling Technology | 1:200 |
| Alexa 488 | Jackson Immuno Research Laboratories Inc. | 1:500 |

**Table S2: Western blotting antibody list**

| **Antibody** | **Supplier** | **Dilution** |
| --- | --- | --- |
| Phospho-Histone H2A.X (Ser139) | Cell Signaling Technology | 1:1000 |
| Poly (ADP-ribose) polymerase (PARP) | Cell Signaling Technology | 1:1000 |
| Caspase-9 | Cell Signaling Technology | 1:1000 |
| Microtubule-associated protein light chain 3 (LC3) | Cell Signaling Technology | 1:1000 |
| Vimentin (R28) | Cell Signaling Technology | 1:1000 |
| Cleaved Caspase-7 (Asp198) (D6H1) | Cell Signaling Technology | 1:1000 |
| β-catenin (D10A8) | Cell Signaling Technology | 1:1000 |
| Cyclin B1 (V152) | Cell Signaling Technology | 1:1000 |
| Caspase-8 (1C12) | Cell Signaling Technology | 1:1000 |
| SQSTM1/p62 (D5L7G) | Cell Signaling Technology | 1:1000 |
| E-cadherin (4A2) | Cell Signaling Technology | 1:1000 |
| N-cadherin (13A9) | Cell Signaling Technology | 1:1000 |
| MMP-2 | Cell Signaling Technology | 1:1000 |
| MMP-9 | Cell Signaling Technology | 1:1000 |
| Cyclin A (H-432) | Santa Cruz Biotechnology | 1:1000 |
| Sox2 | Santa Cruz Biotechnology | 1:1000 |
| c-Myc | Santa Cruz Biotechnology | 1:1000 |
| β-actin | Santa Cruz Biotechnology | 1:3000 |
| TBX3 | Cloud-Clone Corp | 1:1000 |
| Horseradish peroxidase (HRP)-conjugated goat anti-rabbit | Biorad | 1:3000 |
| HRP-goat anti-mouse | Biorad | 1:3000 |
